# Microbiome analysis reveals *Microcystis* blooms endogenously seeded from benthos within wastewater maturation ponds

**DOI:** 10.1128/aem.01585-23

**Published:** 2023-12-20

**Authors:** C. S. Romanis, V. J. Timms, D. J. Nebauer, N. D. Crosbie, B. A. Neilan

**Affiliations:** 1 University of Newcastle, School of Environmental and Life Sciences, Callaghan, Australia; 2 ARC Centre of Excellence for Synthetic Biology, Callaghan, Australia; 3 Melbourne Water, Docklands, Victoria, Australia; University of Delaware, Lewes, Delaware, USA

**Keywords:** *Microcystis*, wastewater treatment, microbiome, metagenomics, microcystin, cyanoHABs

## Abstract

Toxigenic *Microcystis* blooms periodically disrupt the stabilization ponds of wastewater treatment plants (WWTPs). Dense proliferations of *Microcystis* cells within the surface waters (SWs) impede the water treatment process by reducing the treatment efficacy of the latent WWTP microbiome. Further, water quality is reduced when conventional treatment leads to *Microcystis* cell lysis and the release of intracellular microcystins into the water column. Recurrent seasonal *Microcystis* blooms cause significant financial burdens for the water industry and predicting their source is vital for bloom management strategies. We investigated the source of recurrent toxigenic *Microcystis* blooms at Australia’s largest lagoon-based municipal WWTP in both sediment core (SC) and SW samples between 2018 and 2020. Bacterial community composition of the SC and SW samples according to 16S rRNA gene amplicon sequencing showed that *Microcystis* sp. was dominant within SW samples throughout the period and reached peak relative abundances (32%) during the summer. The same *Microcystis* Amplicon sequence variants were present within the SC and SW samples indicating a potential migratory population that transitions between the sediment water and SWs during bloom formation events. To investigate the potential of the sediment to act as a repository of viable *Microcystis* cells for recurrent bloom formation, a novel *in-vitro* bloom model was established featuring sediments and sterilized SW collected from the WWTP. Microcystin-producing *Microcystis* blooms were established through passive resuspension after 12 weeks of incubation. These results demonstrate the capacity of *Microcystis* to transition between the sediments and SWs in WWTPs, acting as a perennial inoculum for recurrent blooms.

**IMPORTANCE:**

Cyanobacterial blooms are prevalent to wastewater treatment facilities owing to the stable, eutrophic conditions. Cyanobacterial proliferations can disrupt operational procedures through the blocking of filtration apparatus or altering the wastewater treatment plant (WWTP) microbiome, reducing treatment efficiency. Conventional wastewater treatment often results in the lysis of cyanobacterial cells and the release of intracellular toxins which pose a health risk to end users. This research identifies a potential seeding source of recurrent toxigenic cyanobacterial blooms within wastewater treatment facilities. Our results demonstrate the capacity of *Microcystis* to transition between the sediments and surface waters (SWs) of wastewater treatment ponds enabling water utilities to develop adequate monitoring and management strategies. Further, we developed a novel model to demonstrate benthic recruitment of toxigenic *Microcystis* under laboratory conditions facilitating future research into the genetic mechanisms behind bloom development.

## INTRODUCTION

Wastewater treatment plants (WWTPs) incorporate microbial activity to metabolize nutrients such as nitrogen and phosphorous. Biological degradation of organic compounds is attributed to a core WWTP microbiome composed of Proteobacteria, Bacteroidetes, Chloroflexi, Actinobacteria, Planctomycetes, and Firmicutes (1–3). These core functional consortia are supplemented by transient and latent bacterial species that reside within WWTPs. Of these, cyanobacteria are a prevalent phyla (4, 5) which consume large quantities of phosphorous and nitrogen during cellular proliferation (6, 7).

Phyla preference for eutrophic conditions, such as those found within waste stabilization ponds (WSPs), often results in the proliferation of potentially toxigenic cyanobacteria species. Potentially toxigenic cyanobacteria, such as *Microcystis,* may outcompete favorable phytoplankton species, reducing the efficiency of the treatment process (4). Further, temperatures exceeding 30°C favour fast-growing *Microcystis* over competing cyanobacterial genera such as *Oscillatoria* and can lead to an abundance of potentially toxigenic species within the WWTP (8). If not properly managed, the production of the intracellular hepatotoxin microcystin by *Microcystis* can endanger the health and safety of those exposed to the recycled water produced (9–11).

**Fig 1:**
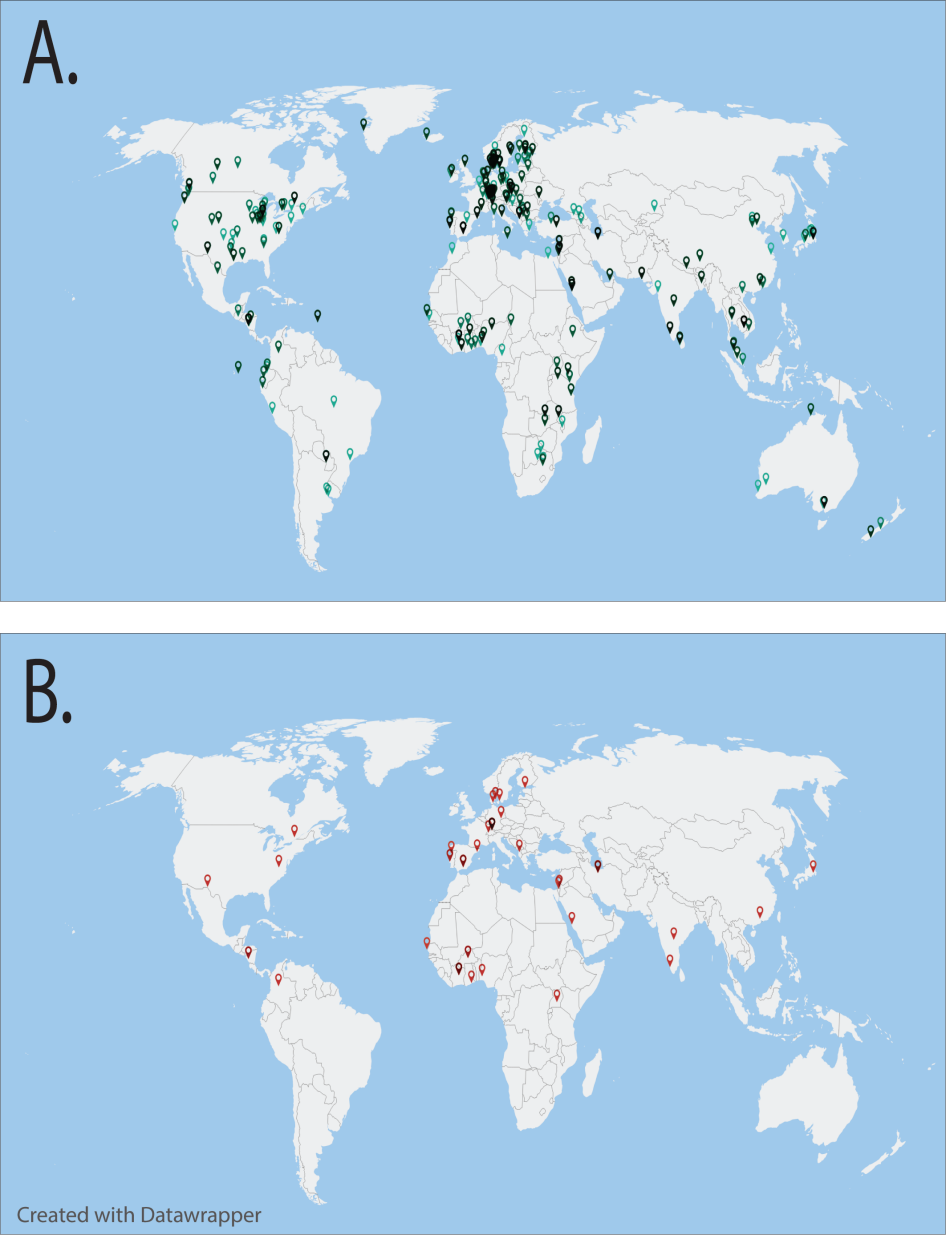
Global distribution of cyanobacterial (**A**) and Chroococcales (**B**) occupancy global wastewater treatment plants. Data were collected by mining all publicly available wastewater metagenomes (*n* = 182, June 2021) on the EMBL MGnify database http://www.ebi.ac.uk/metagenomics

*Microcystis* occupancy of WWTPs is a global phenomenon (Fig. 1) with dense surface proliferations often reported during the summer months (12–15). Warm temperatures promote stabilization of the water column, which favor buoyant *Microcystis* colony formation (16, 17). High-density surface blooms then impede solar penetration of the water column leading to a seasonal reduction in the treatment efficiency of UV sterilisation. In freshwater systems, recurrent seasonal *Microcystis* blooms are often initiated by fluvial seeding from upstream sources (18). Within WWTPs, blooms could be seeded from the bottom sediment/sludge layer, similar to seeding from the benthos of eutrophic lakes (19–25).

*Microcystis* is a meroplanktonic genus that annually transitions through a period of overwintering in the sediment, reintroduction into the water column during spring, pelagic bloom formation in summer and then subsequent resettling of cells to the sediment during autumn (21, 22, 26–28). *Microcystis* cells remain metabolically active and viable within the sediment (29) and capable of producing microcystins during this time (30). In WWTPs, hydrogen peroxide (H_2_O_2_) can be used as an environmentally benign option for the removal of *Microcystis,* which results in a proportional reduction in microcystins that avoids the production of toxic by-products (31). However, treatment efficiency is reduced with depth, and research suggests that H_2_O_2_ is ineffective at eliminating microcystin itself (32). Potentially toxigenic *Microcystis* localized to the sediment may be unaffected and thus available for bloom (re)establishment. Therefore, while treatment with H_2_O_2_ will decrease the cyanobacterial and microcystin loading in the SWs (33) of WWTP lagoons, bloom recurrence may be linked to *Microcystis* re-inoculation from the WWTP lagoon sediment to the WWTP lagoon pelagic zone. This study evaluated the potential for overwintering *Microcystis* to act as a benthic inoculum for subsequent blooms in the maturation ponds of a municipal WWTP.

Melbourne Water’s Western Treatment Plant (WTP), the largest of its kind in Australia, has operational wastewater lagoons that cover an area of 1,746 hectares. The L25W treatment lagoon comprises 10 waste stabilization ponds (lagoons) of varying volumes in series with an average total summer hydraulic retention time of 23.7 days. Pond 3 (L25WP3) is the largest of these ponds with an average hydraulic retention time of 9.2 days and is downstream of an activated sludge plant (ASP) which discharges to the preceding pond (L25WP2). The L25W ASP was commissioned in September 2004 to facilitate microbially driven reduction in the concentration of organic compounds. To capture bloom emergence patterns, the microbiome of the sediment and pelagic compartments of L25WP3 were characterized using 16S rRNA amplicon sequencing of samples collected from 2018 to 2020. The viability of overwintering *Microcystis* to establish colonial surface proliferations was investigated using an *in-vitro* bloom propagation model.

## RESULTS

To assess if the sediments within a municipal wastewater maturation pond act as an endogenous seeding source for perennial cyanobacterial blooms, 36 (SC) samples were collected during winter (2018) and summer (2019 and 2020). Corresponding SW samples were collected from the same time point (*n* = 20). An observable cyanobacterial bloom was reported during each summer collection period which was absent during the winter. L25WP3 is the largest maturation pond onsite and collected water quality parameters, including historical data are available in the Appendices (Appendices; Figure A1). Sequencing of the 36 sediment and 20 water samples from pond L25WP3 yielded 8,676,548 high quality, non-chimeric sequence reads. The median read frequency per sample was 121,895 (range 9,327–460,691) and 48,241 amplicon sequence variants (ASVs) were identified. Rarefaction analysis resulted an upward alpha metric curve showing that the diversity present in all samples was adequately captured (Appendices; Figure A2). ASVs indicated an average of 2396 (96–5,099) observed features in the SC samples and 1111 (199–2,483) within the SW samples.

### Cyanobacterial composition within the WWTP

*Microcystis* (Appendices; Figure A3) was the most abundant cyanobacterial genera, which primarily localized to the SW samples. In the summer of 2019 and 2020, *Microcystis* (>63.5%) dominated all water samples and the highest proportion was seen during February 2019 (0.2–63.5%) and February 2020 (3.0–23.4%), coinciding with the onset of observable bloom formation (Figure A3). The large variation in abundance in February 2019 is due to one sample with a low relative abundance of *Microcystis* (0.2%), likely derived from erroneous sampling that failed to capture the cyanoHAB biomass. Negligible *Microcystis* presence was detected throughout the remaining sampling period (January = 0.04%, July = 0.0%, and October = 0.4%) (Figure A3). *Microcystis* abundance within the sediment follows a different trend; the greatest mean relative frequency in the sediment occurred in January (0.346%) prior to successive decreases in mean relative frequency (February = 0.02%, July = 0.1%, and October = 0.04%). Despite a high degree of variation within the cyanobacterial population of the SC, consideration of the entire bacterial consortia revealed an overall higher composition similarity and stability than the SW samples.

**Fig 2:**
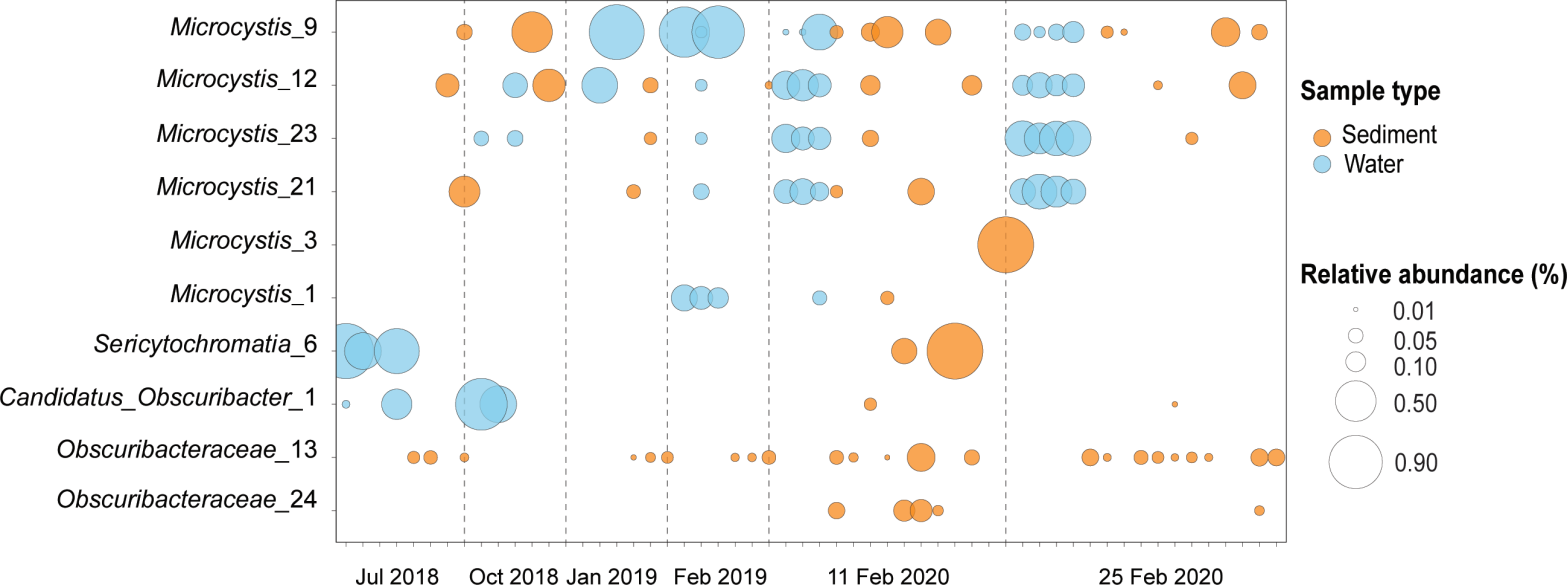
Relative abundances of cyanobacterial community composition of SC (*n* = 36) and SW (*n* = 20) samples. Relative abundance of the 16S rRNA gene was determined using a rarefied frequency-feature table. ASVs were classified to the genus level using the SILVA database (v138). *Microcystis* ASVs demonstrate a temporal relationship where taxa that are abundant in the sediment during the winter are subsequently abundant in the water column during the following summer.

ASVs initially identified as cyanobacteria using the SILVA classifier (*n* = 282) were validated through placement within the Cydrasil reference phylogeny resulting in 170 unique cyanobacteria taxa (Appendices; Table A1). The most abundant ASVs included six *Microcystis*, three Obscuribacteraceae, and one *Sericytochromatia* (Fig. 2). The most abundant ASV, *Microcystis*_9 was identified within the SC samples every year with the highest average relative abundance recorded in winter (2018; 0.08%), with negligible presence in the summer of 2019 and an increase in relative abundance in the summer of 2020 (0.04%) (Fig. 2). *Microcystis*_9 was also detected in the SW samples during the summer sampling periods (2019: 0.3% and 2020: 0.2%) (Fig. 2; Table 1). Two more *Microcystis* ASVs (*Microcystis*_12 and *Microcystis*_21) showed a similar trend in having the highest SC relative abundance in the winter, followed by the highest SW relative abundance in summer (Fig. 2; Table 1). Conversely, *Sericytochromatia*_6 had the highest SC relative abundance in the summer (2020: 0.05%) and the highest SW relative abundance in the winter (2018: 0.29%). Obscuribacteraceae ASVs had a consistent presence in the SC samples (0.002–0.03%) (Fig. 2; Table 1) with *Candidatus_Obscuribacter*_1 having a notable SW proliferation in 2019.

**TABLE 1:** Average relative abundance of the top 10 cyanobacterial ASVs identified from the SC and SW samples of the WTP. Samples collected in 2018 correspond to winter (July and October)^*a*^

ASV	2018		2019		2020	
	SC	SW	SC	SW	SC	SW
*Microcystis*_9	0.08	0.00	0.0005	0.30	0.04	0.19
*Sericytochromatia*_6	0.00	0.29	0.00	0.00	0.05	0.00002
*Microcystis*_12	0.06	0.02	0.01	0.07	0.02	0.15
*Microcystis*_23	0.00	0.008	0.006	0.01	0.005	0.23
*Microcystis*_21	0.04	0.00	0.007	0.01	0.01	0.18
*Candidatus_Obscuribacter*_1	0.002	0.10	0.0004	0.14	0.003	0.0001
*Microcystis*_3	0.00	0.00	0.00	0.00	0.04	0.00
Obscuribacteraceae_13	0.01	0.001	0.02	0.00	0.03	0.003
*Microcystis*_1	0.00	0.00	0.00	0.05	0.002	0.02
Obscuribacteraceae_24	0.00	0.00	0.0004	0.00	0.02	0.003

^*a*^ Samples collected in 2019 and 2020 are reflective of summer (January and February).

**Fig 3:**
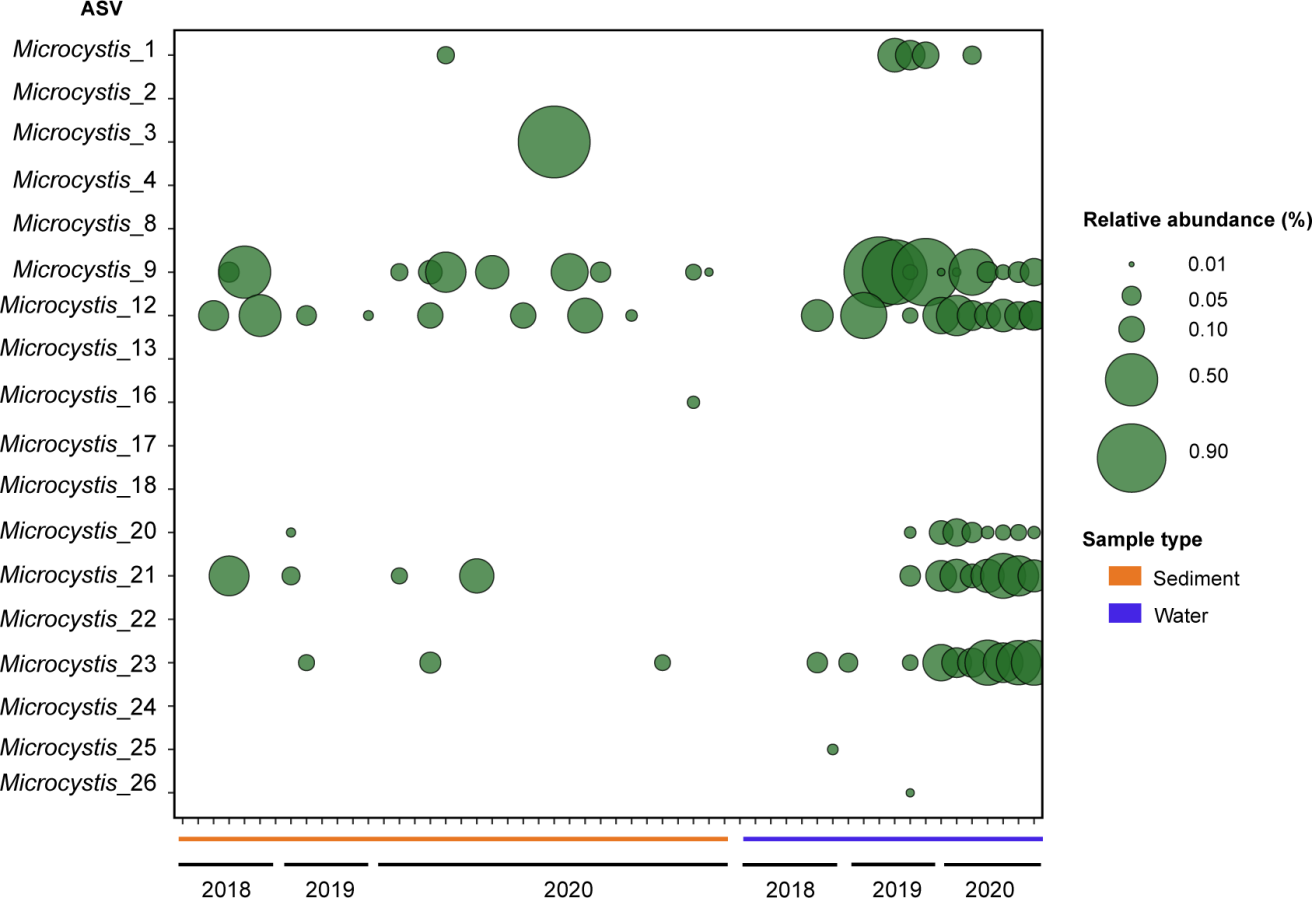
Relative abundances of most abundant *Microcystis* ASVs identified within the SC (*n* = 36) and SW (*n* = 20) samples. Relative abundance of the 16S rRNA gene was determined using a rarefied frequency-feature table. ASVs were classified to the genus level using the SILVA database (v138). *Microcystis* ASVs 9,12, 21, and 23 were all abundant in the sediment during the winter and absent in the summer. The same ASVs were inversely sparse in the water column during the winter and more abundant during the summer.

Consideration of all *Microcystis* ASVs (*n* = 26) shows a consistent pattern of seasonal transference between the SC and SW samples (Fig. 3). The highest average relative abundance for *Microcystis* ASVs occurred in winter (2018) for SC samples (0.007%) and in summer (2020) for SW samples (0.03%). Six *Microcystis* ASVs (*Microcystis*_1, 9, 12, 20, 21, 22, and 23) comprised the bulk of the visible surface blooms that were observed at the WTP in the summer’s of 2019 and 2020 (Fig. 3).

### Cyanobacterial diversity within sediment and water samples

Five alpha diversity metrics were used to determine differences in the cyanobacterial composition between the SC and SW samples (Table 2). Significant differences in cyanobacterial richness and diversity between the SC and SW were only detected when considering Faith’s Phylogenetic Diversity and the number of observed features (Kruskal-Wallis; Faith: *q* = 0.02, observed ASVs: *q* = 0.04). Differences in cyanobacterial composition were less significant than differences in the total bacterial consortia between sample types, where significant differences were detected between the SC and SW samples for the latter, when considering all five alpha diversity metrics (Appendices Table A2). For the total bacterial consortia, the SC samples were significantly richer and more diverse compared to the SW samples (Kruskal-Wallis; Shannon: *P* = 0.0002; Faith: *P* = 0.01; observed ASVs: *P* = 0.0001; Pielou *P* = 0.007; Chao1: *P* = 0.0004). The SC samples clustered tightly and showed higher bacterial composition similarity (38.26%) compared to the SW samples (20.71%). SIMPER analysis determined that *Microcystis* was the most influential taxa contributing to similarity within the SW samples. No cyanobacteria species were implicated in contributing to variation between the SC samples (Table 3).

**TABLE 2:** Kruskal-Wallis pairwise group comparisons of cyanobacterial communities using five alpha diversity indexes^*a*^

	Sediment core versus surface water
Shannon’s Index	0.207712
Faith’s Phylogenetic Diversity	0.016436
Number of Observed Features	0.036721
Chao1 Index	0.737018
Pielou’s Evenness	0.781207

^*a*^ *q* values (Benjamin and Hochberg corrected *P* values) are shown.

**TABLE 3:** SIMPER analysis identifying the percentage contribution of the top 10 ASVs influencing dissimilarity between the SC and SW samples^*a*^

ASV	Represented phyla	% Contribution to dissimilarity	% Average abundance SC	% Average abundance SW
*Microcystis*	Cyanobacteria	4.92	0.004	0.08
*Pseudomonas*	Proteobacteria	2.8	0	0.04
*Flavobacterium*	Bacteroidota	2.63	0	0.04
*Escherichia-Shigella*	Proteobacteria	2.41	0.03	0
*Clostridium*	Firmicutes	2.22	0	0.03
Anaerolineaceae	Chloroflexi	1.89	0.03	0
*Denitratisoma*	Proteobacteria	1.75	0.03	0
*Sulfuricurvum*	Campilobacterota	1.57	0.03	0
Gallionellaceae	Proteobacteria	1.47	0.02	0
Sporichthyaceae	Actinobacteria	1.46	0	0.02

^*a*^ Similarity percentage analysis was performed on the Bray-Curtis dissimilarity metric between SC and SW sample groups.

**Fig 4:**
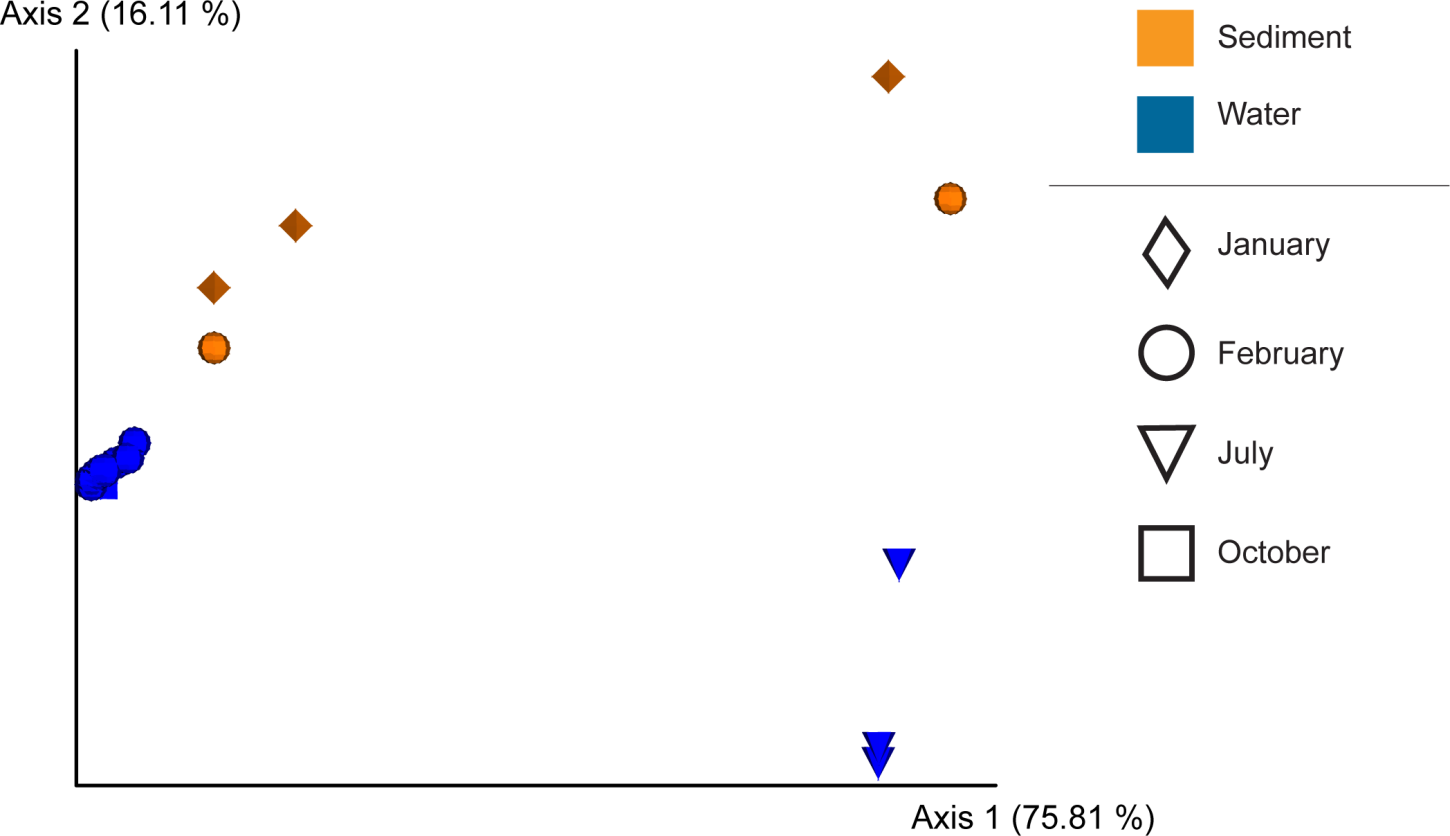
PCoA plot of weighted UniFrac distances of cyanobacterial communities from SC (orange) and SW (blue) samples from the Western Treatment Plant. Samples were collected during Summer (January-diamond, February-circle) and Winter (July-triangle, October-square).

PCoA identified two dimensions defining 92% of the compositional differences between the cyanobacterial populations within the SC and SW samples (PERMANOVA: *q* = 0.032). The SC samples have a cyanobacterial similarity composition of 11.5%, lower than the SW samples (17.6%). The SW samples also displayed a much tighter clustering pattern except for samples collected in July 2018 (Fig. 4). Six ASVs, four *Microcystis*, one *Sericychromatia*, and one Obscuribacteraceae, are cumulatively responsible for 93.6% of compositional differences in the cyanobacterial populations between the SC and SW samples (Table 4). *Microcystis* contributed 4.9% to the total 86% variation of the bacterial composition between the SC and SW samples.

**TABLE 4:** SIMPER analysis identifying the percentage contribution of cyanobacterial ASVs influencing dissimilarity between the SC and SW samples^*a*^

ASV	% Average Abundance SC	% Average abundance SW	% Contribution to dissimilarity	Cumulative % contribution to dissimilarity
*Microcystis_*9	0.04	0.18	19.12	19.12
*Microcystis_*12	0.02	0.09	13.88	33.00
*Sericytochromatia*_6	0.03	0.11	12.04	45.04
*Microcystis*_23	0.004	0.10	11.54	56.58
*Microcystis*_21	0.02	0.08	9.18	65.76
Candidatus_Obscuribacter_1	0.004	0.06	5.70	71.46

^*a*^ similarity percentage analysis was performed on the Bray-Curtis dissimilarity metric between SC and SW sample groups.

### Propagation of *in-vitro Microcystis* blooms producing microcystin

**Fig 5:**
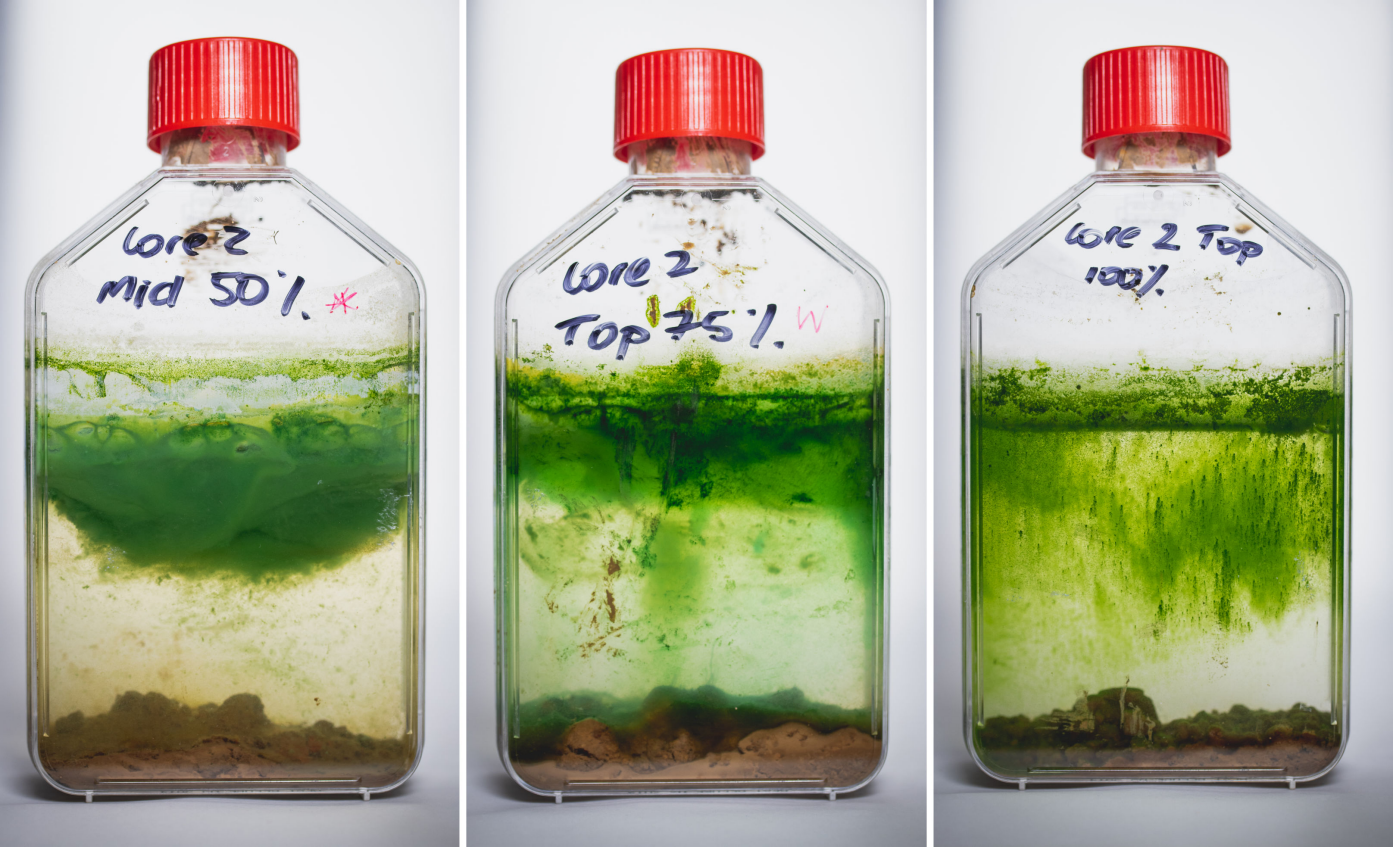
*in-vitro* bloom propagation chambers at 16 weeks. Media was prepared by supplementing filtered surface water from L25W-P3 with 50%, 75%,or 100% BG11 media. Chambers were inoculated with ~12 g of L25W-P3 sediment. Cultures were maintained under continuous fluorescent light (25 µM photons m^−2^ s^−1^) and at a temperature of 24°C.

**Fig 6:**
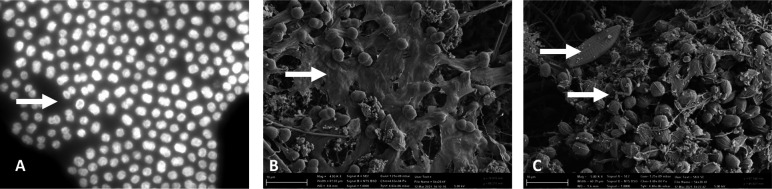
Fluorescence and scanning electron micrographs of *in-vitro* cyanobacterial blooms. Fluorescence micrograph (**A**) captured at 400× total magnification on Zeiss Axioskop fluorescence phase contrast microscope. Scanning electron micrographs generated using the Zeiss Sigma VP scanning electron microscope at 5,000× total magnification on 50% BG11 (**B**) and 75% BG11 *in-vitro* bloom material (**C**). Arrows in (**A**) and (**B**) indicate extracellular polymeric mucilage. Arrows in (**C**) highlight non-cyanobacterial bacteria in association of bloom development. Scale bars in each image indicate 10 µm.

The viability of *Microcystis* bloom formation arising from benthic inoculation was investigated through the conception of *in-vitro* propagation models. Sediment collected from the WTP was incubated with filtered site water supplemented with varying concentrations of BG-11 media. Single-celled, dispersed cyanobacteria were observed within the 100% BG-11 and 75% BG-11 chambers within 6 weeks and in 50% BG-11 after 7 weeks (Appendices; Figure A4). However, once established after 8 weeks, cellular growth was more rapid in 50% BG-11 compared to 100% BG-11 and 75% BG-11 conditions. Dense cellular agglomeration bound within a mucilage matrix, as seen in *in-situ Microcystis* blooms, was observed in both 50% BG-11 and 75% BG-11 (Fig. 5). *Microcystis* cell aggregates bound in mucilage matrix were morphologically identified through autofluorescence (Fig. 6A). The *Microcystis* aggregate was observed using scanning electron microscopy (SEM) (Fig. 6B and C) and showed algal-bacterial flocs encased in extracellular polymeric substances. Phylogenetic analysis of the 16S rRNA gene sequences indicated that each of the *in-vitro* surface blooms was predominantly comprised of Proteobacteria, Cyanobacteria, and Bacteroidota (Figure A3). At the genus level, the *in-vitro* blooms housed an unclassified cyanobacteria (x̄ = 38.1%), *Microcystis* (x̄ = 11.0%), *Rickettsiales* (x̄ = 24.2%), *Kapabacteriales* (x̄ = 5.1%), and *Brevundimonas* (x̄ =4.3%). Species diversity and richness of the *in vitro* bloom were significantly different to the sediment samples (*q* = 0.02), but no difference in bacterial composition was found between the *in vitro* bloom and the SW samples (*q* = 0.5).

### Detection of microcystin production by *in-vitro Microcystis* bloom

**Fig 7:**
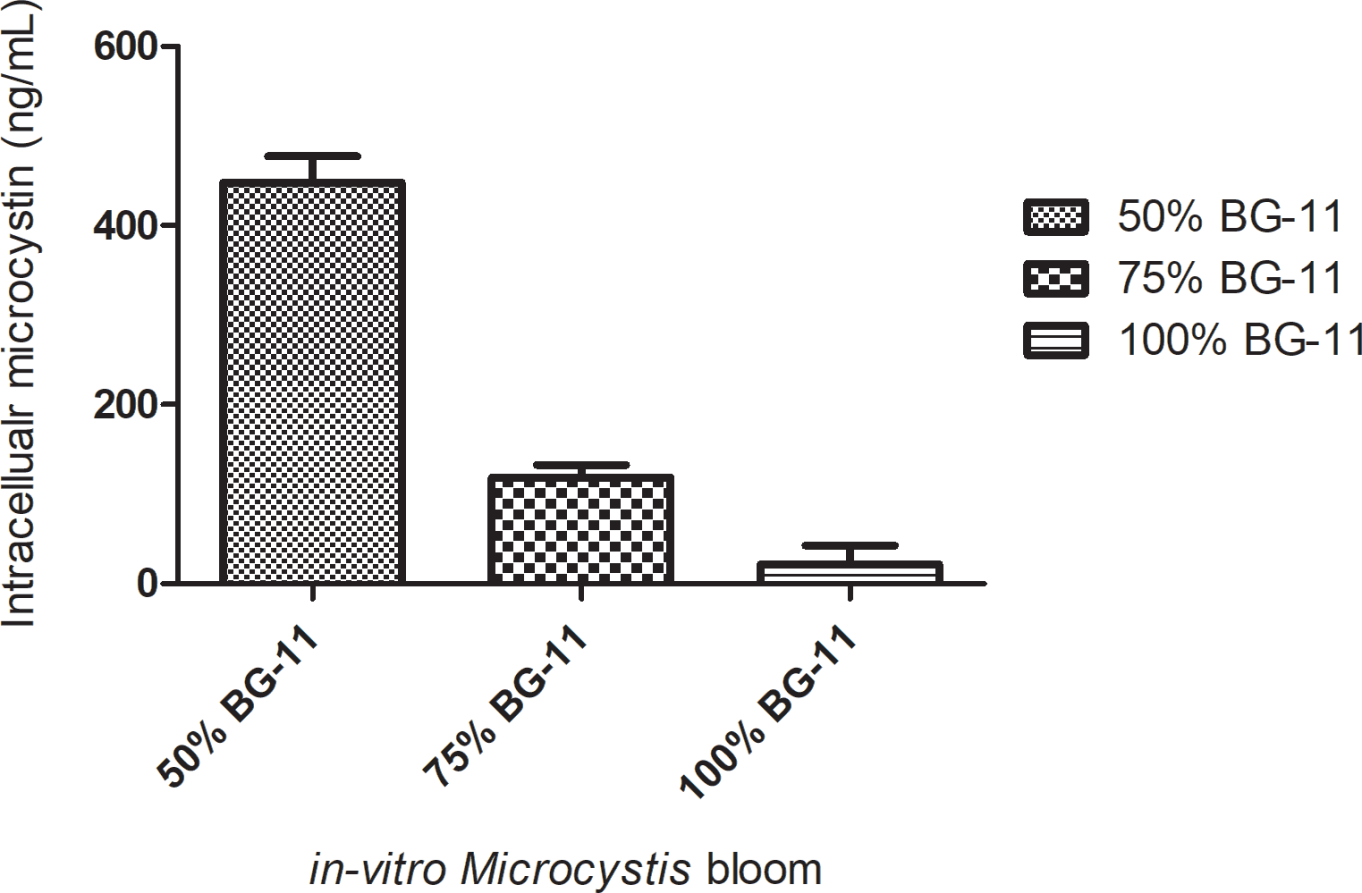
Total microcystin and nodularin concentration detected by ELISA in 1 mL of each *in-vitro* bloom. Total MC/NOD concentration (ng/mL) was determined for each propagation condition; 50% BG-11, 75% BG-11, and 100% BG-11 by sampling the surface bloom material. ELISA performed in replicate (*n* = 2). Microcystin concentration was inversely correlated to nutrient concentration.

Intracellular microcystins were detected in all *in-vitro* cyanoHABs in concentrations inverse to the amount of BG-11 media used to supplement the propagation chambers (Fig. 7). The cyanoHAB established under 50% BG-11 enrichment produced the highest concentration of microcystins, 447.73 ng/mL, compared to those under 75% BG-11 (118.99 ng/mL) and 100% BG-11 (21.68 ng/mL) conditions. Similarly, microcystin production was confirmed within all *in-vitro* cyanoHABs using LC-MS/MS. Peaks corresponding to MC-LR were observed in all MS^2^ spectra at *m/z* 995.5560 at retention time 22.72 (Appendices; Figure A5). The strongest peak observed across all samples corresponds to the PhCH_2_CHOMe fragment of the ADDA moiety at *m/z* 135. Additional fragments commonly assigned to MC-LR, *m/z* 599, *m/z* 553, and *m/z* 163 were detected in all samples. The *in-vitro* cyanoHAB from 100% BG-11 did not contain MC-LR fragments assigned at *m/z* 470 or *m/z* 570 which were present in the MS^2^ spectra for 50% BG-11 and 75% BG-11 (Appendices; Table A3).

## DISCUSSION

In wastewater treatment facilities, *Microcystis* blooms significantly impede water treatment yet the underlying community dynamics of bloom-forming *Microcystis* remains unclear. We paid particular attention to the cyanobacterial population and the potential for migratory behavior using 16S rRNA gene sequencing. High-throughput sequencing has emerged as a tool for monitoring wastewater systems; however, most studies focus on the bacterial composition of activated sludge (34–36) rather than the lagoon-based consortia in our study. To characterize temporal dynamics, the cyanobacterial community composition of the sediment and SW of the largest maturation pond at a WWTP was profiled. The same *Microcystis* ASVs were identified within both the sediment and pelagic samples suggesting that recurrent and potentially toxigenic *Microcystis* blooms can arise from benthic seeding in WWTP. Propagation of *in-vitro Microcystis* blooms from WTP SCs supports the hypothesis of endogenous bloom inoculation.

The degree to which the genotypic diversity of *Microcystis* occupying the benthos may influence the composition and genomic variability of SW proliferations is largely unclear (37). The potential for sediments to act as a genetic repository for perennial bloom development should be of concern to water quality managers and the greater limnological community as adaptations to environmental stimuli may determine bloom severity and toxicity (38–40). The cyanobacterial community residing with the Western Treatment Plant is considerably diverse with 170 unique ASVs detected across the sampling period. The most abundant of which was *Microcystis* correlating with previous microscopy-based site observations conducted by the water utility. Seasonal shifts in the relative abundance of *Microcystis* were observed at both the genus and ASV levels indicating that seasonal migration of cyanobacteria occurs at the WTP.

The highest percentage of *Microcystis* reads in SC samples was recorded in January (0–0.4%) consistent with previous work that saw sedimentation occurring in the summer months as a consequence of increased carbohydrate accumulation (20). The proportion of *Microcystis* in February 2020 (0–0.2%), coincided with substantial *Microcystis* presence observed in the water column. While a decrease of only 0.2% of *Microcystis* reads was observed in the sediment samples, previous studies suggest that only a small percentage (<1%) of sedimentary *Microcystis* is required to re-inoculate the water column and develop into seasonal and potentially toxigenic proliferations (41). The persistence of cyanobacteria in the benthos has long been associated with the problematic recurrence of cyanobacterial harmful algal blooms (cyanoHABs) (42–46). Therefore, it is critical that a greater understanding of benthic processes and factors impacting the transition between planktic and benthic lifestyles is established.

After the bloom collapse in February, the relative abundance of *Microcystis* within sediments decreased over winter (July 0.1% to October 0.06%) and suggests a resettling of cells post bloom. *Microcystis* was identified in only one SW sample during this time, in October 2018 (0.4%). This potentially represents the beginning of water column reinvasion by viable *Microcystis* cells as the water temperature increases during the summer. Bloom decline is initiated by changes in environmental conditions and internal factors, such as a reduction in nutrient and light availability (47). After bloom collapse, the distribution and sedimentation of benthic *Microcystis* are dependent on hydraulic components such as water body movement and depth (48).

Evidence that *Microcystis* cells deposited within the benthos perform an inoculative role is found within the cyanobacterial community at an ASV level. Throughout the study period, 26 *Microcystis* ASVs were observed to have temporal variation in relative abundance within the sediment and SW samples (Fig. 3; Table 1.) Potential benthic seeding is seen from *Microcystis*_9 that was absent from the water column in winter 2018 but present in the sediment (0.08%) in the same year. In the following summer, the relative abundance of *Microcystis*_9 in the sediment decreased (0.005%) but was detected in the water column (0.3%). A period of bloom collapse followed in the winter of 2019; therefore, sedimentation of *Microcystis*_9 cells could be responsible for the increase in the relative abundance detected in 2020. Similar transitions may be observed for *Microcystis*_12 and *Microcystis*_21 in 2019. While rates of *Microcystis* sedimentation vary throughout the bloom period, settling rates are >50 magnitudes greater during bloom collapse than in other periods of bloom formation or persistence (49). Irradiance type also plays a role in *Microcystis* sedimentation, e.g., exposure to UVB radiation (280–320 nm) induces high sedimentation rates, particularly of non-toxic strains (50). These natural sedimentation processes likely result in viable cells persisting within the WTP benthos. Once a *Microcystis* ASV was detected within the sediment it would remain, albeit with varying relative abundance in the sediment for each consecutive year of sampling. *Microcystis*_1 was first detected in 2019 in the SWs and was absent from the sediment samples of 2018 and 2019. After a period of pelagic growth, the ASV was subsequently detected in the sediments the following year. This pattern occupancy is suggestive of cyclical bloom formation within the WTP. ASVs detected in the sediment were not always prevalent in the water column with *Microcystis*_9 and *Microcystis*_12 abundant in 2019, whereas *Microcystis*_21 and *Microcystis*_23 were more prominent in 2020 despite all samples being detected in 2018. This is in line with previous research showing that *Microcystis* recruitment from the sediment was viable after 3 years (28). The reintroduction of Microcystis cells to the water column is more dependent on environmental conditions and inferred growth rate, while the size of the inoculum is not as pivotal (27). In addition to benthic inoculation, benthic accumulation is also particularly troublesome for water authorities as active toxin production may still occur within the sediment (51). Industrial processes such as coagulation and flocculation through the use of agents such as PAC + Sepiolite, may be considered by water utilities wishing to reduce cell viability within the sediment, limiting inoculation stock for future blooms (52). To test the viability of benthic toxigenic *Microcystis* to act as an inoculum for the development of pelagic blooms, *in-vitro* propagation chambers were established with controls established without a sediment seeding source. The seasonal vertical migration of *Microcystis* is thought to be either an active or passive process. Passive resuspension involves the re-entry of *Microcystis* cells into the water column facilitated by wind induced turbulence or bioturbation (29, 53). Conversely, active recruitment is governed by cellular mechanisms such as increased gas vesicle production, changes to colony structure, and physiological responses to increases in water temperature (46, 54). In WWTPs, the effects of passive resuspension may be limited by an absence of vertebrate species; however, the often shallow systems are typically vulnerable to vertical mixing of the water column by wind (55). During the propagation experiments, unicellular *Microcystis* cells re-entered the water column. The irregular morphologies of the *Microcystis* cells were observed using scanning electron microscopy and revealed colonies encased in a thick mucilage layer of extracellular polymeric substances (Fig. 6). The mechanism for *Microcystis* colony formation under environmental conditions is unknown (56); however, it is thought to confer advantages for bloom formation, affording them increased defense against predation (57, 58) and improved responsiveness to high light, compared to free-living cells (59). Typically, colony morphology studies have limited application in the *in-situ* management of water utilities as under laboratory conditions *Microcystis* exist as single cells (60, 61). The abrupt onset of *Microcystis* blooms in eutrophic systems is not linked to rapid cellular proliferation but rather vertical migration of *Microcystis* colonies at the water’s surface (62). A previous investigation on a *Microcystis* bloom in Lake Taihu showed that intermittent turbation through wind or wave action promoted dense *Microcystis* colony formation (63) while light attenuation, nutrient availability and cellular release of microcystin play an avid role in colony maintenance (64–66). In the absence of any turbation mechanism or alteration of abiotic factors, we identified the *in-vitro* blooms as non-axenic, implying that colony formation may also arise as a morphological response to cosmopolitan growing conditions. Several studies have documented the occurrence of heterotrophic bacteria residency within cyanobacterial bloom biomass (67–69). Furthermore, non-axenic *Microcystis aeruginosa* will synthesize and secrete extracellular polysaccharides (EPS) upon introduction to heterotrophic species (70, 71). The formation of EPS may act to tether and stabilize *Microcystis* cells together and to additional species in mutually beneficial colonies. Sequencing of the *in-vitro* blooms confirmed a cosmopolitan composition dominated by *Microcystis* and an unclassified cyanobacterium. *Brevundimonas, Kapabacteriales*, and *Rickettsiales* also predominated and *Brevundimonas* is known as a heterotrophic denitrifying species critical to the remediation of wastewater (72). The species diversity and richness between the *in-vitro* blooms and the naturally occurring *Microcystis* blooms of the WWTP were not significantly different (*q* = 0.5) suggesting the propagation models are an adequate laboratory solution to study bloom formation dynamics in the future.

Previous studies have estimated that only a small proportion of viable *Microcystis* cells are required to inoculate the water column during cyanoHAB formation (30). The passive *in-vitro* inoculation of the water column by *Microcystis* cells cannot be considered an axenic process; the presence of Proteobacteria within the bloom material experiment is consistent with earlier hypotheses that benthic seeding coincides with concurrent recruitment of sediment-associated microbes (73–75). Intracellular microcystins were detected in all of our *in vitro* cyanoHABs. The ecological role of microcystin biosynthesis by *Microcystis* species transitioning from sediment hibernation to water column inoculation is complex (76, 77). Toxin biosynthesis may fulfil various roles, including allelopathic (78, 79), iron chelation (80, 81), intra-species communication (82, 83), defensive roles (84, 85), involvement in photosynthesis (86, 87), and adaption to oxidative stress (88, 89). The high concentration of intracellular microcystins detected within the *in-vitro* cyanoHAB propagated using 50% BG-11 suggests that toxin production is linked to nutrient availability and cellular density. This is consistent with previous observations that microcystin-producing strains are more resilient to nutrient deprivation (88), and while microcystin concentrations were normalized per mL of *in-vitro* propagation sample in this study, future experiments normalized with respect to biomass will help clarify this conclusion.

Temperature was not a factor in the recruitment of *Microcystis* genotypes as each *in-vitro* bloom propagation chamber was kept under a constant temperature of 24°C. MC-LR biosynthesis is inversely correlated with temperature, where toxin production optimally occurs at 18–28°C, significantly lower than the temperature required for optimum cellular growth (33°C) (90–92). This suggests that toxin production may occur prior to visible colony detection and that future research using the *in-vitro* blooms will ascertain toxin synthesis during each phase of benthic recruitment. Water quality will continue to be vulnerable to *Microcystis* proliferation and microcystin presence as climate change leads to elevated SW temperatures in enclosed systems such as lakes (93). Higher temperatures will result in an increase in *Microcystis* cell concentrations, and consequently an increase in the genetic machinery required for microcystin biosynthesis as toxic genotypes have a higher growth rate at elevated temperatures compared to non-toxic genotypes (17). Additionally, toxic genotypes also generate a higher concentration of EPS (66) which may trap organic material and particulate matter. Filtration apparatus may become blocked as EPS-bound particulate adheres to the filter surface reducing treatment capacity at utilities that employ this method of remediation (94). The establishment of successful *in-vitro* cyanoHAB propagation chambers will enable research into the physiological role of microcystin generation in *Microcystis* bloom formation and resuspension from the sediment. While the data herein suggest that benthic seeding may contribute towards the development of perennial blooms at the WTP, amplicon-based studies lack the resolution to adequately differentiate *Microcystis* strains owing to high-sequencing similarity and ambiguous taxonomic delineation (95). Studies incorporating high resolution technologies such as whole metagenome sequencing are required to validate the endogenous inoculation within maturation ponds.

### Conclusions

WWTPs are often frequented by seasonal *Microcystis* cyanoHABs that decrease water treatment efficacy and efficacy. Recurrent blooms are likely seeded from a population of viable *Microcystis* cells overwintering in the sediment. Our *in-vitro* cyanoHAB propagation model demonstrated the migratory and cosmopolitan nature of developing *Microcystis* blooms and established that the WTP sediment acts as a benthic repository for endogenous seeding of pelagic blooms.

## MATERIALS AND METHODS

### Study area and field sampling

The Western Treatment Plant (37°59′07.5″S, 144°37′36.1″E) is located approximately 35 km south-west of Melbourne City and directly adjacent to Port Philip Bay. A total of 36 SCs were collected opportunistically on 31/7/2018, 13/02/2019, and 25/02/2020 in quadruplicate from L25WP3 using polyvinyl chloride pipe which was speared into the sludge creating a self-sealing plug. Lagoon water incidentally captured was drained from the samples before being immediately stored on ice. The pelagic bacterial composition of L25WP3 was captured as 250 mL SW grab samples collected in fortnightly increments from 2018 to 2020. SW samples (*n* = 20) were collected in quadruplicates and preserved with the addition of lugol iodine solution while on site. Three additional SW samples were collected in February 2020 without fixation and stored on ice for culturing experiments. All samples were transported on ice overnight to the University of Newcastle. Upon arrival, the fixed SW samples were filtered onto 0.22 µm glass microfiber filters (Filtech, Australia) and stored at −30°C. A portion of the SC samples were retained on ice for the establishment of *in-vitro* propagation models, while the remainder were stored at −30˚C without fixative agents.

### Nucleic acid extraction

Genomic DNA was extracted from 500 mg (wet weight) of the homogenized SC samples and the SW filter samples using the protocol described by Nercessian et al. (96) with minor modifications. The sample and 1.3 g of lysis beads (0.6 g 0.1 mM zirconia-silica beads, 0.6 g 0.7 mM zirconia beads, and 0.1 g 3 mM glass beads) were suspended in 500 µL CTAB-extraction buffer (10% CTAB in 1.6 M NaCl and 240 mM Kaliumphosphate buffer pH 8.0), to which 50 µL 10% SDS and N-Lauroylsarcosin were added. Phase separation was performed using phenol-chloroform-isoamyl alcohol (25:24:1), and homogenization (FastPrep FP120, Thermo Savant), followed by another round using chloroform-isoamyl alcohol (24:1). Nucleic acids were precipitated overnight using 30% PEG 6000–1.6 M NaCl at 4˚C. Pellets were washed in 70% ethanol and resuspended in 50 µL diethyl pyrocarbonate (DEPC) treated water.

### Sequencing and data analysis

Sequencing was performed by the Ramaciotti Centre for Genomics, University of New South Wales, Australia using the universal primer pair 27F/519R (Table 5) for amplicon sequencing of the V1-V3 region of the 16S rRNA gene (2 × 300 bp PE). Sequencing was performed on a MiSeq (Illumina, San Diego, USA) sequencing platform using the MiSeq Reagent Kit v3 (Illumina) at. De-multiplexed sequencing data are available on the NCBI Sequence Read Archive (SRA) under project PRJNA987429. The 16S rRNA V1-V3 amplicon sequencing files were pre-processed, quality filtered, and analyzed using the Quantitative Insights Into Microbial Ecology (QIIME) analysis package 2 (v2020.11) (97). Owing to the lower quality of the reverse reads, only the forward read was used for analysis. The QIIME2-DADA2 plugin (98) was used on the 12,010,394 raw reads to perform quality filtering (PHRED score <34), remove chimeras, trim the first five bp of each read and truncate to 240 bp. This resulted in 8,676,549 sequence reads and 48,241 ASVs across 56 samples. Distribution of sequence reads amongst individual samples is available in Appendices, Table A.

**TABLE 5:** PCR amplification primers and thermocycler settings used in this study^*a*^

Primer	Sequence	Reference
27Fl	AGA GTT TGA TCC TGG CTC AG	(99)
519r	GTA TTA CCG CGG CKG CTG	(100)

Temperature (°C)	Duration	Number of cycles
94	3 min	1
94	30 s	30
55	30 s	
72	1 min	
72	7 min	1
20	Hold	**-**

^*a*^ Each PCR reaction contained: 1× Tris-HCl buffer, 2.5 mM MgCl2, 1.5 mM dNTP mix, 1 mM BSA, 0.2 U taq (Bioline), and 10 pmol of Forward and Reverse primers.

### Bacterial community diversity analysis

Phylogenetic diversity metrics were produced using the QIIME2 q2-phylogeny plugin. This generated an MAFFT masked alignment (101) of the 48,241 ASVs which was used as input for phylogenetic tree construction using the maximum-likelihood method in FastTree (v2.0.0) (102). Generation of alpha- and beta-diversity metrics was performed on the constructed phylogenetic tree using the QIIME2 q2-diversity plugin. Outlier effects of rare taxa were limited by rarefying to a depth of 124,870 sequences per sample using the QIIME2 diversity alpha-rarefaction command (Appendices, Figure A2). To perform alpha-diversity assessment, Shannon, Faith’s-PD, Observed features, Chao1, and Pielou’s evenness indices were measured using the rarefied sequences (Appendices, Table A5). Associations between alpha-diversity metrics and sample type were calculated using pairwise Kruskal-Wallis analysis in the QIIME2 package. Weighted UniFrac and Jaccard distance matrices were generated to assess beta-diversity measures. Principal coordinate analysis (PCoA) was performed on each distance matrix to determine factors driving community dissimilarity. Pairwise permutational multivariate analysis of variance (PERMANOVA) (103) was used to assess the effects of sampling data on community structure. To ascertain the species percentage contribution between water and sediment samples, analysis of similarity percentages (SIMPER) (104) was conducted using PRIMER 7 (v7.0.13) (105).

### Taxonomic assignment of bacterial communities from L25WP3

Taxonomic assignment of 48,241 ASVs was performed using a Naïve Bayesian classifier in QIIME2. The classifier was tailored to the 16S rRNA gene V1-V3 hypervariable region targeted by the universal primer pair 27F/519r (Appendices, Table 5). The classifier was trained using the non-redundant small subunit SILVA reference database clustered at 99% sequence similarity (v138) (106) and tested using the representative sequences from the QIIME2 “Moving Pictures” tutorial (v2019.10, https://docs.qiime2.org/2020.11/tutorials/moving-pictures/). The representative sequence reads from each ASV putatively identified as cyanobacteria or chloroplast (*n* = 282) were aligned to the Cydrasil cyanobacterial sequence database (v3) (107) using SSU-ALIGN (v0.1.1) (108). ASVs were placed in the Cydrasil reference tree using EPA-ng (v0.3.8) (109) and those with a cyanobacterial designation like weight ratio of ≤0.4 were removed.

### *in-vitro Microcystis* bloom propagation models

The unfixed SW samples were filtered through a 0.22-µm glass microfiber membrane (Filtech, Australia) and enriched with BG-11 to generate culturing media (BG-11:SW) in the following ratios: 100:0 (100% BG-11), 75:25 (75% BG-11), and 50:50 (50% BG-11). Approximately 12 g of the fresh SC was transferred into a sterile T-175 tissue culture flask with a polyethylene vented cap, before the addition of 200 mL BG-11:SW culturing media. To simulate a decreasing light gradient with depth, aluminium foil was used to cover the bottom third of the flask. Each propagation flask was established in triplicate and placed in a constant light and temperature room of 24°C and 25 µM photons m^−2^ s^−1^ light penetration. Control flasks were established without the sediment inoculum. *Microcystis* cell density was quantified at 630X magnification using fluorescence phase contrast microscopy (Zeiss Axioskop) with a hemocytometer. At this resolution, enumeration of single cells within the colonies was achieved.

Taxonomic characterization of the bacterial composition of the *in-vitro Microcystis* blooms was conducted through the amplicon sequencing of the hypervariable regions V1-V3 of the 16S rRNA gene. Approximately 1 mL of *in-vitro* bloom material from each propagation condition was filtered onto 0.22 µm glass microfiber filters. Nucleic acid extraction, DNA sequencing, and bioinformatics analysis were performed using the methods described above.

### Scanning electron microscopy analysis of *in-vitro* bloom propagation models

A 5 mL surface bloom sample from the densest cyanobacterial harmful algal bloom (cyanoHAB) (models 50% BG-11, 75% BG-11) was filtered onto 0.22 µm glass microfiber filters and fixated using glutaraldehyde 2.5% (vol/vol) in 0.075 M phosphate buffer for 30 min. The membrane filter was washed three times with 0.075 M phosphate buffer (5 min each) and dehydrated using an ethanol gradient of 10%, 30%, 50%, 70%, and 90% (15 min each), followed by 100% ethanol three times (15 min each). Critical point drying (CPD-30, Balzers, Lichtenstein) for 15 cycles and platinum coasting (SPI-Module Carbon Coater) was done prior to SEM with a Zeiss Sigma VP scanning electron microscope operating at 5.00 kV.

### Detection of microcystins within *in-vitro* propagation models

Intracellular microcystin production within the *in-vitro* cyanoHABs was quantified by microcystin-ADDA enzyme-linked immunosorbent assay (ELISA) (Enzo Life Sciences Inc., NY, USA), following the manufacturer’s instructions. Quantification was performed using 1 mL of surface bloom material from each propagation chamber and analysis was performed in duplicate to account for error and reproducibility. ELISA results were confirmed by a targeted full-scan LC-MS/MS search using a QExactive Plus Orbitrap (QE+) mass spectrometer (Thermo Scientific, MA, USA) in positive ion mode. Extracellular quantification was performed using 2 mL of surface bloom material from each propagation chamber and pelleting through centrifugation at 4000 × *g* for 10 min. Samples were concentrated by adding supernatant to a C18 cartridge (Sep-pak; Waters Association), eluting with 90% methanol and evaporating to dryness in a speed-vac concentrator. Intracellular microcystin was extracted from the pellet with 1 mL 90% methanol, acidified with 1% acetic acid, vortexed, then rotated on a sample mixer for 1 h at room temperature. After centrifugation (10,000 × *g* for 10 min), the supernatant was evaporated in a speed-vac concentrator. Dried extracts were stored at −30°C and dissolved in methanol when required. The Orbitrap QE+ was set up to isolate microcystin precursor targets at *m/z* 995.55 and *m/z* 498.28 and for higher energy collisional dissociation (HCD) fragmentation, which corresponds to MC-LR and its doubly charged ion, respectively. Microcystin targets were inspected manually to confirm identity and fragments were required to be identified within two biological replicates for confidence.

## Data Availability

All raw sequences generated in this paper were deposited in the National Centre for Biotechnology Information (NCBI) Sequence Read Archive (SAR) with the accession number PRJNA987429. Scripts used within this study are available on github; https://github.com/c-romanis/Benthic_migration_paper.git and intermediate files may be accessed at https://doi.org/10.5281/zenodo.10021717.

## SUPPLEMENTAL MATERIAL

Supplemental figures and tables aem.01585-23-s0001.docx Figure A1. Temporal observations of nutrient concentrations within L25WP3 maturation pond at the Western Treatment Plant. Figure A2. Alpha-diversity rarefaction curves for the 56 samples from L25WP3. Figure A3. Relative abundance of bacterial community composition of SW, SC and in-vitro bloom samples Table A2. Kruskal-Wallis pairwise group comparisons for five alpha diversity indexes. Figure A4. Cyanobacterial growth curves and standard deviations for in-vitro bloom propagation chambers. Figure A5: ms2 chromatograms at m/z 995.5560. Table A3. Sample information and sequence pre-processing data. Table A4. ms2 fragments detected in the 50% BG-11, 75% BG-11 and 100% BG-11 in-vitro propagation models used to confirm the structure of MC-LR. Table A5. Diversity indices applied within this study.

Supplemental Table 1 aem.01585-23-s0002.xlsx Table A1. Relative abundance of cyanobacterial ASVs.

ASM does not own the copyrights to Supplemental Material that may be linked to, or accessed through, an article. The authors have granted ASM a non-exclusive, world-wide license to publish the Supplemental Material files. Please contact the corresponding author directly for reuse.
