# Supplementary material for: Microbiome analysis reveals Microcystis blooms endogenously seeded from benthos within wastewater maturation ponds
Source: Appl Environ Microbiol. 2023 Dec 20;90(1):e01585-23. doi: 10.1128/aem.01585-23 (PMC10807444; doi:10.1128/aem.01585-23)
Supplement: Supplemental figures and tables — Figure A1. Temporal observations of nutrient concentrations within L25WP3 maturation pond at the Western Treatment Plant. Figure A2. Alpha-diversity rarefaction curves for the 56 samples from L25WP3. Figure A3. Relative abundance of bacterial community composition of SW, SC and in-vitro bloom samples Table A2. Kruskal-Wallis pairwise group comparisons for five alpha diversity indexes. Figure A4. Cyanobacterial growth curves and standard deviations for in-vitro bloom propagation chambers. Figure A5: ms2 chromatograms at m/z 995.5560. Table A3. Sample information and sequence pre-processing data. Table A4. ms2 fragments detected in the 50% BG-11, 75% BG-11 and 100% BG-11 in-vitro propagation models used to confirm the structure of MC-LR. Table A5. Diversity indices applied within this study. [file aem.01585-23-s0001.docx]

**
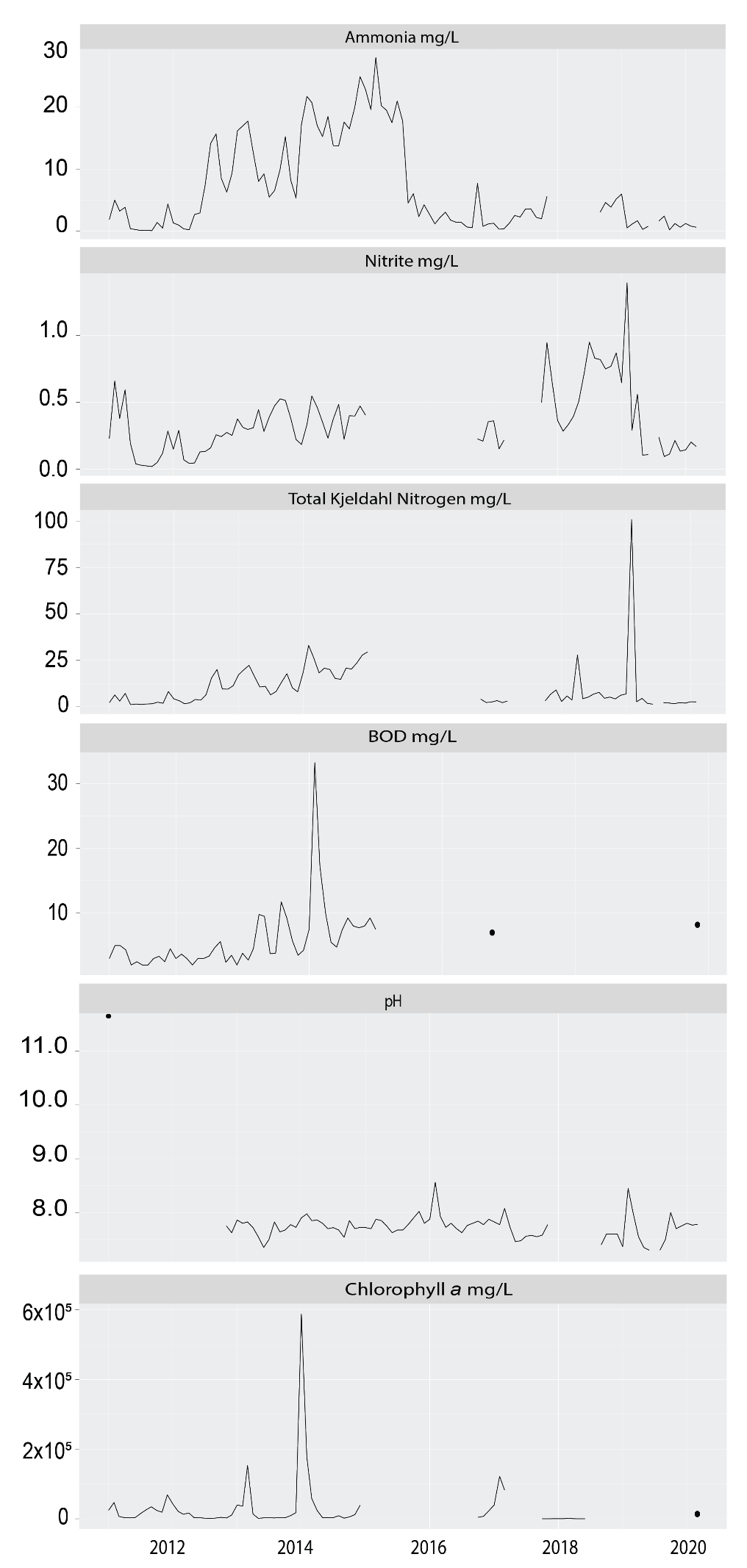
**

**Figure A1. Temporal observations of nutrient concentrations within L25WP3 maturation pond at the Western Treatment Plant.** Metadata was collected by Melbourne Water Corporation on an ad-hock basis from 2012-2020.


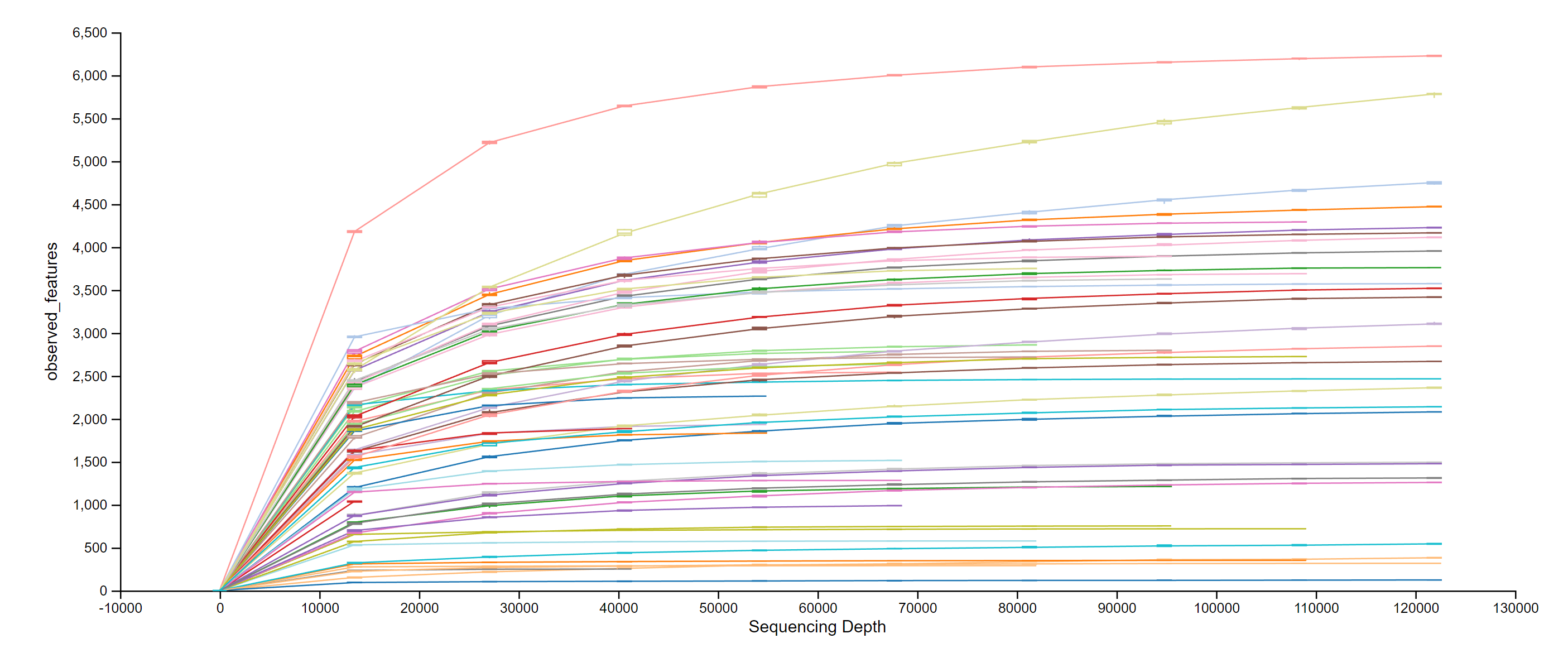


**Figure A2. Alpha-diversity rarefaction curves for the 56 samples from L25WP3.** Detected diversity is plotted against predicted total diversity. The number of sequences per sample is reported as sequencing depth on the x-axis. Community richness is measured through the number of observed features (ASVs) on the y-axis.


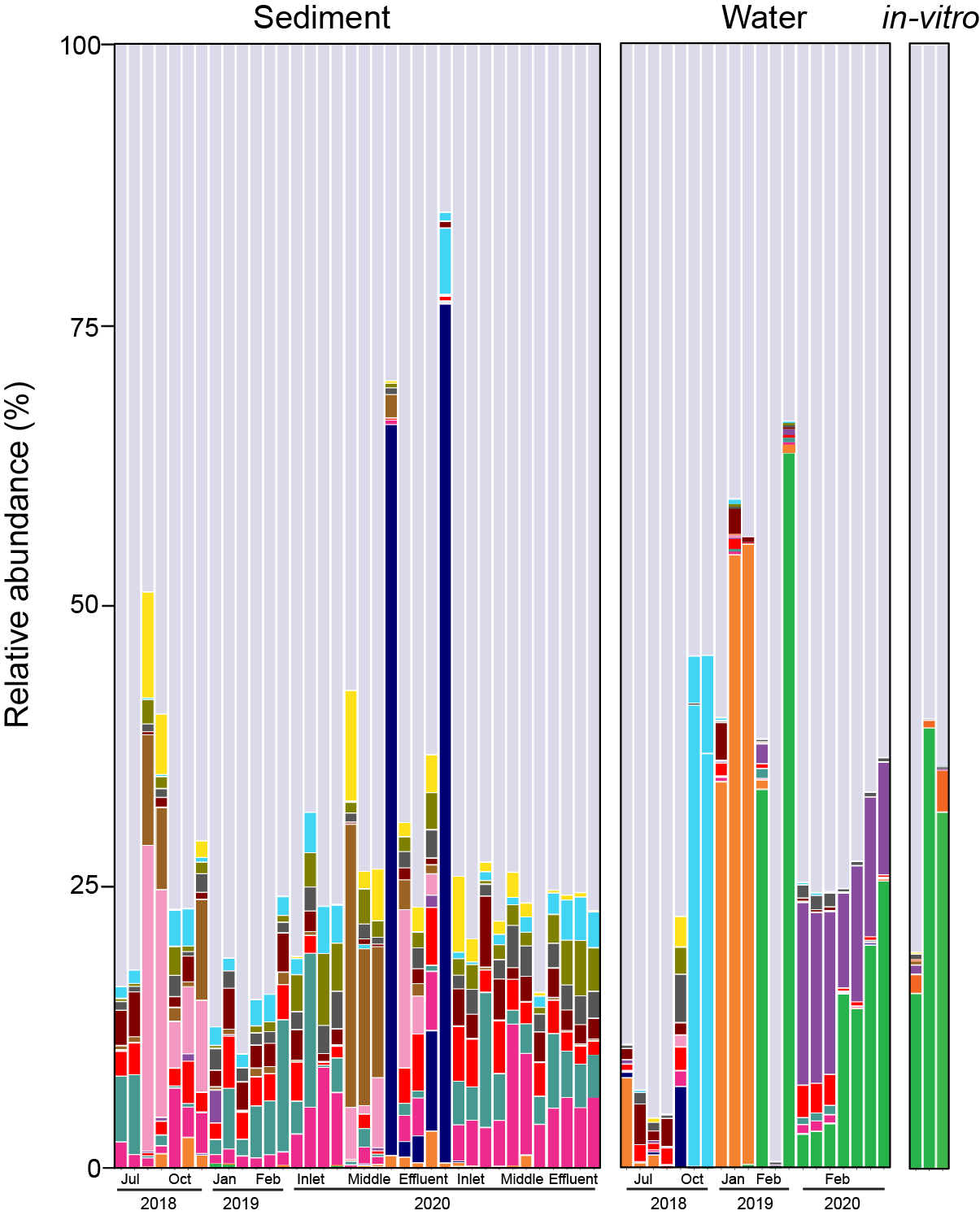

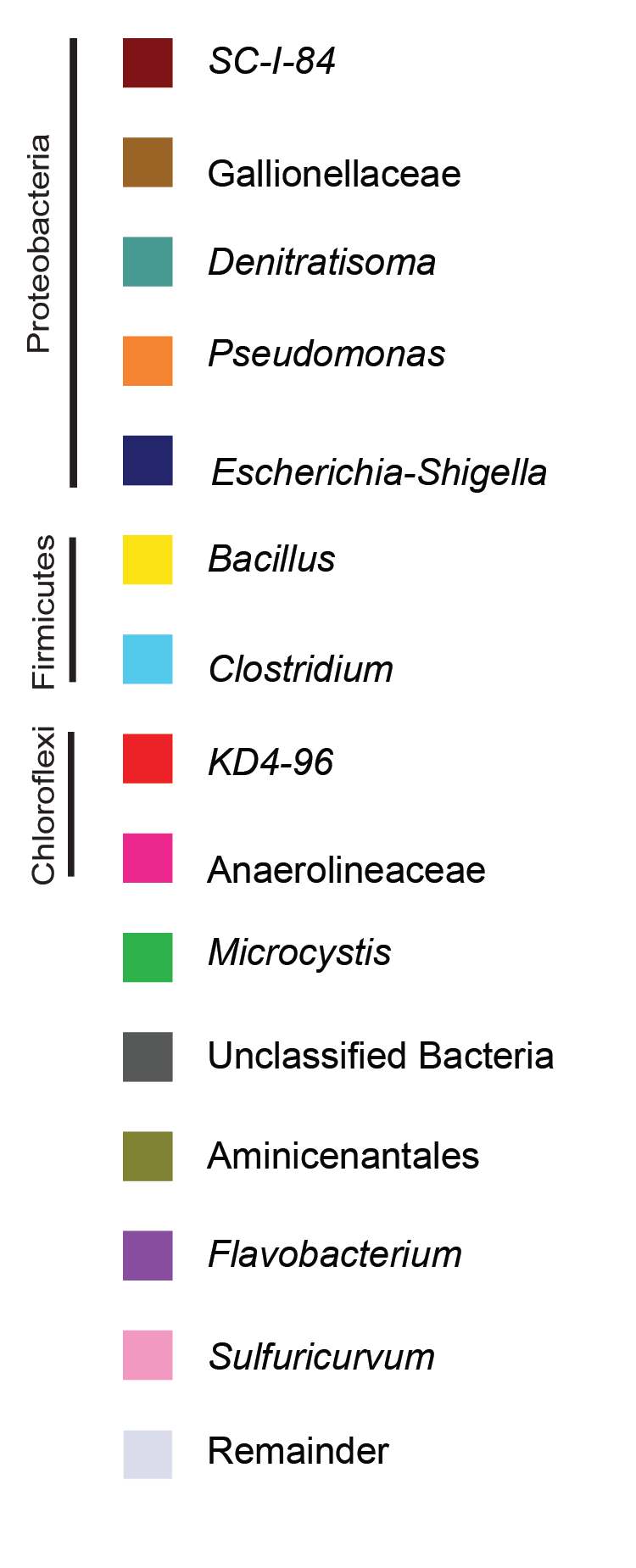


**Figure A3. Relative abundance of bacterial community composition of SW, SC and *in-vitro* bloom samples.** ASVs were classified to the genus level using the SILVA database (v138). All 2020 SC samples were collected on the 11^th^ and 25^th^ of February from the pond inlet, mid-pond or the pond outlet.

**Table A2. Kruskal-Wallis pairwise group comparisons for five alpha diversity indexes. *q-*values (Benjamin and Hochberg corrected *p*-values) are shown.**

|  | **Sediment Core vs. Surface Water** |
| --- | --- |
| Shannon’s Index | 0.000195 |
| Faith’s Phylogenetic Diversity | 0.010941 |
| Number of Observed Features | 0.000132 |
| Chao1 Index | 0.000384 |
| Pielou’s Evenness | 0.007240 |
|  |  |


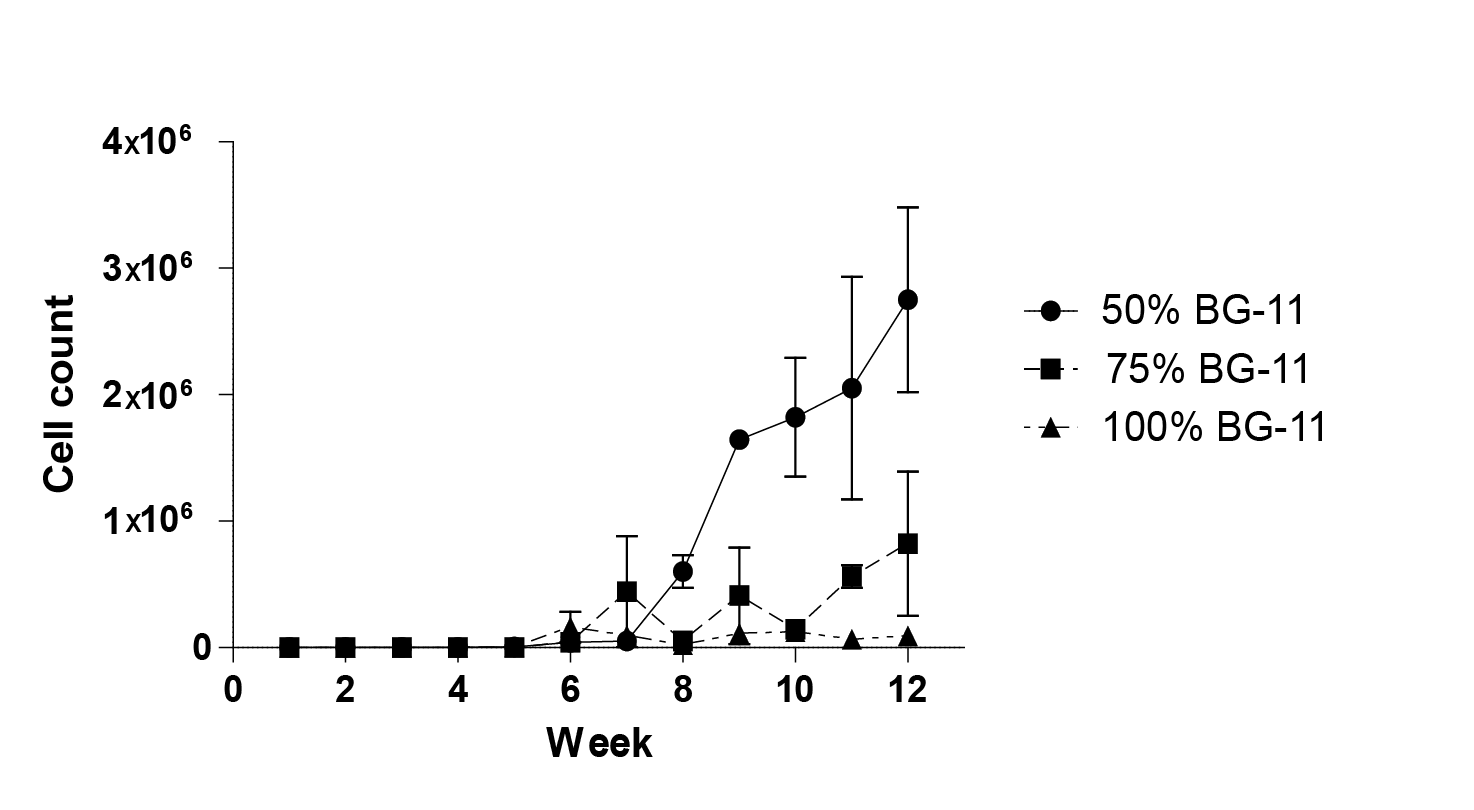


**Figure A4. Cyanobacterial growth curves and standard deviations for *in-vitro* bloom propagation chambers.** Propagation media was generated through supplementing filtered surface water from the WTP with 50%, 75% or 100% BG-11. Propagation chambers were maintained at 24˚C with 25 µM photons m^−2^ s^−1^ light penetration.


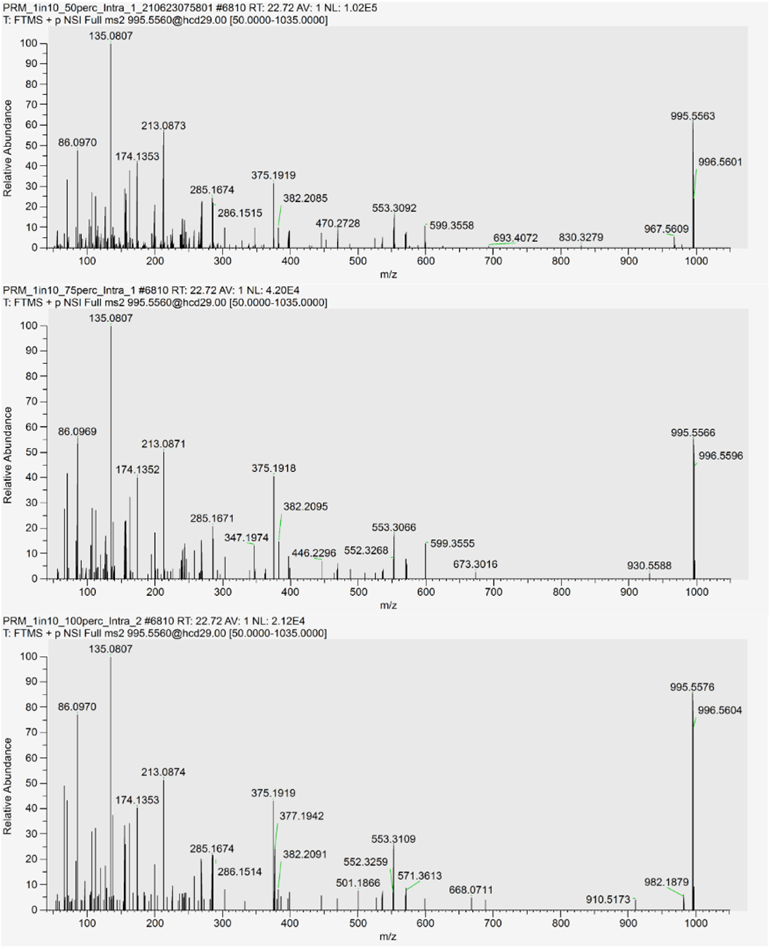


BG11:100

BG11:75

BG11:50

**D**

**D**

**D**

**C**

**C**

**B**

**B**

**B**

**A**

**A**

**A**

**Figure A5: ms^2^ chromatograms at m/z 995.5560.** Chromatograms were collected from material extracted from the BG11:50 (top), BG11:75 (middle) and BG11:100 (bottom) *in-vitro* cyanoHABs. All chromatograms were collected at retention time = 22.72. Peaks corresponding to Microcystin-LR were observed at (**A**) 135 *m/z,* (**B**) 553 *m/z,* (**C**) 599 *m/z* and (**D**) 995 *m/z.*

**Table A3. ms^2^ fragments detected in the 50% BG-11, 75% BG-11 and 100% BG-11 *in-vitro* propagation models used to confirm the structure of MC-LR.** The theoretical m/z is calculated for each corresponding fragment, and the m/z value detected in each sample is shown.

| **MC-LR fragments** | **Theoretical *m/z*** | **Detected *m/z*** | | | | |
| --- | --- | --- | --- | --- | --- | --- |
|  |  | **50% BG-11** | | **75% BG-11** | | **100% BG-11** |
| Arg+Adda+Glu+H | 599 | 599.35 | 599.36 | | 599.36 | |
|  | 470 | 470.27 | 470.27 | | - | |
|  | 570 | 570.34 | 570.33 | | - | |
|  | 553 | 553.31 | 553.31 | | 553.31 | |
| PhCH_2_CHOMe (Adda fragment) | 135 | 135.08 | 135.08 | | 135.08 | |
| Acetyl side-chain | 163 | 163.11 | 163.11 | | 163.11 | |

**Table A4. Sample information and sequence pre-processing data**

| **Sample ID** | **Sample Type** | **Collection Date** | **Raw Reads** | **Filtered** | **Denoised** | **Non-chimeric** |
| --- | --- | --- | --- | --- | --- | --- |
| Sed12Ac | Sediment | 25/02/2020 | 194709 | 122676 | 115885 | 115299 |
| Sed12Bc | Sediment | 25/02/2020 | 390317 | 223704 | 213606 | 208833 |
| Sed12Cc | Sediment | 25/02/2020 | 417136 | 117811 | 116633 | 113697 |
| Sed12D | Sediment | 25/02/2020 | 116476 | 105927 | 97929 | 97394 |
| Sed13A | Sediment | 25/02/2020 | 131521 | 111190 | 104212 | 103249 |
| Sed13B | Sediment | 25/02/2020 | 159005 | 133478 | 127114 | 124987 |
| Sed13C | Sediment | 25/02/2020 | 71518 | 59129 | 54752 | 54197 |
| Sed13D | Sediment | 25/02/2020 | 194917 | 121595 | 115482 | 113738 |
| Sed3A | Sediment | 11/02/2020 | 196539 | 170914 | 163477 | 161815 |
| Sed3B | Sediment | 11/02/2020 | 333741 | 308954 | 301283 | 293658 |
| Sed3C | Sediment | 11/02/2020 | 336074 | 297335 | 291087 | 281403 |
| Sed3D | Sediment | 11/02/2020 | 567205 | 484053 | 472806 | 459350 |
| Sed8Ac | Sediment | 25/02/2020 | 182975 | 44826 | 43927 | 43378 |
| Sed8Bc | Sediment | 25/02/2020 | 17375 | 11679 | 9350 | 9327 |
| Sed8Cc | Sediment | 25/02/2020 | 122218 | 99030 | 90806 | 90495 |
| Sed8Dc | Sediment | 25/02/2020 | 150738 | 84831 | 80394 | 79804 |
| SedC1c | Sediment | 11/02/2020 | 198324 | 184509 | 180114 | 172929 |
| SedC2c | Sediment | 11/02/2020 | 210156 | 163395 | 157041 | 152316 |
| SedC3c | Sediment | 11/02/2020 | 213405 | 177595 | 171855 | 165250 |
| SedC4c | Sediment | 11/02/2020 | 574468 | 222639 | 221480 | 219365 |
| SedD2c | Sediment | 11/02/2020 | 153842 | 89533 | 87356 | 86032 |
| SedD3c | Sediment | 11/02/2020 | 316170 | 177316 | 173816 | 168212 |
| SedD4c | Sediment | 11/02/2020 | 323514 | 88090 | 87328 | 87033 |
| SedE1 | Sediment | 4/10/2018 | 137336 | 122860 | 117327 | 114438 |
| SedE3 | Sediment | 4/10/2018 | 76576 | 62146 | 59252 | 58202 |
| SedE4c | Sediment | 4/10/2018 | 127075 | 71938 | 70981 | 69104 |
| SedF1 | Sediment | 31/07/2018 | 187923 | 144304 | 137388 | 136401 |
| SedF2 | Sediment | 31/07/2018 | 85533 | 74204 | 70236 | 69660 |
| SedF3c | Sediment | 31/07/2018 | 173867 | 157796 | 151518 | 147362 |
| SedF4c | Sediment | 31/07/2018 | 69349 | 60124 | 55622 | 55106 |
| SedG1c | Sediment | 22/01/2019 | 69354 | 48868 | 45938 | 45675 |
| SedG2 | Sediment | 22/01/2019 | 101023 | 76988 | 73152 | 72916 |
| SedG3 | Sediment | 22/01/2019 | 196255 | 175840 | 167728 | 166966 |
| SedH1 | Sediment | 13/02/2019 | 244719 | 155929 | 150530 | 149744 |
| SedH2 | Sediment | 13/02/2019 | 232433 | 182108 | 175650 | 174367 |
| SedH4 | Sediment | 13/02/2019 | 116065 | 98416 | 94306 | 93507 |
| Sed1T2 | Water | 4/10/2018 | 102736 | 80573 | 78918 | 78236 |
| Sed1T3 | Water | 4/10/2018 | 13397 | 10338 | 9463 | 9463 |
| Sed2T1 | Water | 31/07/2018 | 370614 | 298825 | 294823 | 290196 |
| Sed2T2 | Water | 4/10/2018 | 129432 | 100117 | 99132 | 96458 |
| SedD4 | Water | 29/01/2019 | 265044 | 115580 | 114826 | 113537 |
| SedD5 | Water | 29/01/2019 | 598993 | 487772 | 485551 | 458932 |
| SedD6 | Water | 29/01/2019 | 173514 | 144831 | 144125 | 134231 |
| SedE6 | Water | 11/02/2019 | 298752 | 207292 | 205840 | 197986 |
| SedE7 | Water | 11/02/2019 | 653029 | 468887 | 467421 | 460691 |
| SedE8 | Water | 11/02/2019 | 284690 | 201097 | 198815 | 187465 |
| SedS1T3 | Water | 31/07/2018 | 239759 | 131970 | 128646 | 121895 |
| SedS2T1 | Water | 31/07/2018 | 185096 | 84691 | 83785 | 82536 |
| SedS2T2 | Water | 31/07/2018 | 204162 | 169342 | 165548 | 163424 |
| WCE1 | Water | 10/02/2020 | 254990 | 160297 | 155893 | 154033 |
| WCE3 | Water | 10/02/2020 | 160505 | 102365 | 98564 | 97513 |
| WCE4 | Water | 10/02/2020 | 37774 | 27921 | 26019 | 25776 |
| WCF10 | Water | 25/02/2020 | 106609 | 83457 | 81729 | 80249 |
| WCF7c | Water | 25/02/2020 | 198088 | 136448 | 134373 | 131892 |
| WCF8 | Water | 25/02/2020 | 152093 | 105835 | 104059 | 102540 |
| WCF9 | Water | 25/02/2020 | 191266 | 137887 | 135668 | 133578 |

**Table A5. Diversity indices applied within this study**

| **Diversity Index** | **Purpose of Assessment** | **Reference** |
| --- | --- | --- |
| **Alpha Diversity** | | |
| Shannon | Quantitatively determine the richness and evenness of each sample | (Shannon 1948) |
| Faith’s Phylogenetic Diversity | Assessment of community richness that includes phylogenetic relationships between features | (Faith 1992) |
| Pielou’s evenness | Determine how well each species is represented | (Pielou 1966) |
| Chao1 | Measure species richness with deference to low-abundance data | (Chao 1984) |
| **Beta Diversity** | | |
| Weighted UniFrac | Quantitative measure of community dissimilarity calculated to include the weighting of phylogenetic tree branches based on ASV abundance | (Lozupone et al. 2007) |
| Jaccard | Qualitative measure of community dissimilarity | (Jaccard 1901) |
